# Avalanche effect for chemically modified dust mitigation from surfaces

**DOI:** 10.1038/s41598-020-80811-2

**Published:** 2021-01-12

**Authors:** Johnny Ebaika Adukwu, Bekir Sami Yilbas, Almaz Jalilov, Hussain Al-Qahtani, Ahmet Z. Sahin, Abdullah Al-Sharafi, Abba Abdulhamid Abubakar, Mubarak Yakubu, Mazen Khaled, Ghassan Hassan

**Affiliations:** 1 Mechanical Engineering Department, King Fahd University of Petroleum and Minerals, Dhahran, 31261 Saudi Arabia; 2 K.A. CARE Energy Research and Innovation Center at Dhahran, Dhahran, Saudi Arabia; 3 Center of Research Excellence in Renewable Energy (CoRE-RE), KFUPM, Dhahran, 31261 Saudi Arabia; 4 Chemistry Department, KFUPM, Dhahran, 31261 Saudi Arabia; 5 Chemistry and Earth Sciences Department, Qatar University, 2713 Doha, Qatar

**Keywords:** Energy science and technology, Engineering, Mechanical engineering

## Abstract

Cost effective dust mitigation from surfaces is one of the challenges in various sectors. The reduction of dust adhesion on surfaces plays a vital role for dust mitigation from surfaces under the gravitational influence. Creating an avalanche effect on dusty surfaces improves the dust mitigation rate and provides an effective cleaning process. Hence, solution treatment of dust by low concentration hydrofluoric acid is considered towards reducing dust adhesion on glass surfaces. To increase the rate of dust mitigation, the avalanche influence is created by the higher density and larger size particles (5300 kg/m^3^ and ~ 50 µm) than the average size dust particles (2800 kg/m^3^ and 1.2 µm) via locating them in the top region of the dusty glass surfaces. Mitigation velocity of the dust particles is evaluated using a high-speed recording system and the tracker program. Findings revealed that solution treatment (curing) of the dust particles results in the formation of fluorine compounds, such as CaF_2_ and MgF_2_, on dust surfaces, which suppress dust adhesion on surfaces. OSHA Globally Harmonized System lists the fluorine compounds formed as environmentally non-harmful. Avalanche's influence results in dust mitigation at a smaller tilt angle of the glass surface (~ 52°) than that of the case with none-avalanche influence (63°). Area cleaned on the glass surface, via dust mitigation, is larger as the avalanche is introduced, which becomes more apparent for the solution treated dust particles. Dust mitigation under avalanche influence improves optical transmittance of the dusty glass samples by a factor of 98%.

## Introduction

Dust on solid surfaces affects the surface properties while creating performance degradation of devices, such as solar energy harvesting panels. Recent changes in atmospheric behavior result in severe environmental conditions including large scale dust settlements on surfaces. Current and future clean energy production policies aim to utilize natural and renewable energy resources. One of the abounded natural sources is the Sun, which can be used for creating electricity via solar-photovoltaic and solar-thermal applications. In solar-photovoltaic applications, the thickness of the dusty layer becomes critical for device performance, in such case, dusty layer absorbs, scatters and diffuses incoming solar radiation while reducing solar radiation reaching on the active surfaces. Hence, dust mitigation from active surfaces becomes vital for the efficient operation of photovoltaic panels. Different methods have been suggested and implement for dust mitigation, such as water cleaning, air-jet blowing, brushing, water droplet rolling, etc.^1,2^. All the suggested cleaning methods require external power to run the cleaning system. Although the method of droplet rolling on the inclined hydrophobic surface is considered as one of the self-cleaning methods for dust mitigation^3^, it involves clean water, which becomes difficult to implement in the areas where clean water is scarce. Utilizing the gravitational potential and avalanche influence for dust mitigation from inclined surfaces is one of the promising methods for dust removal. However, further investigations are needed to explore the minimization of dust residues on the inclined surface after dust mitigation by avalanche influence under the gravitational potential.

Environmental dust has the potential to create various degrading effects in many ways such as in health^4^, agriculture^5^, buildings^6^, mining^7^, renewable energy systems etc.^8^. Prevention of dust scaling and its mitigation from surfaces become one of the recent challenges, particularly in the renewable energy sector. Although several techniques have been adopted for improved mitigation dust from energy harnessing surfaces such as electrostatic shielding^9^ and droplet rolling/sliding^10^; however, cost effective mitigation method(s) is still under research. Introducing public tariff shearing the cost of dust scaling is under study^11^. Nevertheless, adopting different techniques and assessment of dust mitigation methods resulting in efficient and cost effective dust removal is fruitful. Strong adhesion of dust on surfaces prevents the simple techniques to be adopted for dust mitigation from surfaces largely in space explorations^12^. Individual dust particle has high surface roughness parameter (1.5–1.7)^13^, which reduces contact area between the substrate surface settled and dust; yet, because of high surface free energy of dust (~ 120 mN/m^2^^13^) causes strong adhesion of the dust particles on substrate surfaces. This becomes critically important for small size particles because of reduced roughness parameter (smoother surfaces), which increases interfacial (contact area) and enhances particle adhesion on the contacted surfaces^14^. Mimicking the nature for dust removal from inclined surfaces, under gravitational influence, can be utilized for mitigation of dust from surfaces. Moreover, surfaces for renewable energy applications, such as photovoltaic panels, are made from glasses or polycarbonate protective sheets, which are high surface energy materials and adhesion of dust on surfaces becomes stronger. In addition, the dust particles compose of various elements and having different shapes and sizes. Some of the constituting compounds of dust do not yield stoichiometric elemental ratios while possessing ionic forces on dust surfaces, which is particularly the case for small dust particles (< 2.5 µm)^15^. Small particles tend to adhere to strong bonds on the surfaces. Hence, large surface inclinations become a necessity for mitigating these particles under the force of gravity. Although the large dust particles can be mitigated from surfaces after surface inclination, small size dust residues can cover the large area on the substrate surface while reducing the overall optical transmittance. Moreover, hydrophilizing of surfaces is one of the alternative methods reducing dust adhesion on surfaces. However, hydrophilizing involves with surface treatment towards texturing and/or thin layer lower surface energy material coatings, which changes optical characteristics of the surface while degrading the optical transmittance. This has a draw back for solar energy harvesting via photovoltaics because of reduced device performance under low solar power transmittance^16^. On the other hand, one of the innovative approaches is incorporating the chemical modification technique that reduces dust surface free energy and improves dust texture characteristics while lowering dust-solid surface interfacial contact area. Consequently, research into cost effective chemical treatment of dust particles towards dust adhesion minimization on surfaces becomes essential.

Environmental dust particles possess carbonates (CaCO_3_ and MgCO_3_) and salt compounds (NaCl and KCl) and they can react with acid solutions while forming compounds, which may have lower surface energies. In addition, a chemical reaction can cause surface grafting on the dust particles, which reduces the interfacial contact area on the surface settled. The dilute acid treatments, such as diluted hydrofluoric acid, of dust can modify the surface characteristics of the dust particles. However, care must be taken to eliminate the hazardous effect(s) of diluted hydrofluoric acid treatment of dust surfaces. The initial tests revealed that the treatment of dust with a diluted hydrofluoric acid solution gives rise to the main constituting compound of CaF_2_ on the dust particle surfaces. Moreover, CaF_2_ is not listed as a harmful product in accordance with the OSHA Globally Harmonized System (GHS, HCS 2012 (29 CFR 1910.1200)). In addition, CaF_2_ does not contain any hazardous parts/components as stated in section 302 EHS TP (Emergency Planning Extremely Hazardous Substance Threshold Planning Quantity, 40 CFR 355). Consequently, dust treatment towards reducing surface free energy and altering surface texturing, via chemical grafting, becomes fruitful through incorporating the diluted hydrofluoric acid solution. Initially, diluted hydrofluoric acid treatment tests were conducted towards achieving the reduced surface energy and grafting the dust particle surfaces. In addition, several tests were conducted to evaluate the diluted hydrofluoric acid treatment on the PV panel protective cover surface in terms of chemical (compound formations), mechanical (pit site formations on the surface), and optical (transmittance and absorption) degradations on the cover surfaces. Since the protective surface has polycarbonate cover, no degradation of surface properties is observed after diluted hydrofluoric acid treatment. Moreover, mitigating chemically treated dust from smooth surfaces, by gravitational potential, requires the inclination of dusty surfaces. To increase the rate of dust mitigation creating the avalanche influence becomes favourable. The avalanche influence can be generated via locating the additional heavier particles (higher density than the dust particles) at the top region of the inclined dusty surfaces^2^. As the inclination angle increases, the gravitational force overcomes the pinning force of large size particles, which creates rolling/sliding of these particles on the inclined surface. Small size particles, which are on the pathway of large particles in motion, are pushed down by the large size heavy particles while generating avalanche phenomenon on the inclined surface^17^. The size effect of avalanching particles causes the segregation of large and small particles via kinetic sieving^18^ and squeeze expulsion^19^, which is associated with the occupation of small particles in between large particles while levering large particles on surfaces during rolling/sliding motion. This allows large size particles occupying in the top region of the surface of the dust layer while small size dust remains below the large size particles in the bottom region of the dust layer. The occupation of dust particles can settle on the inclined surface and create residues, which lower the optical transmittance of the inclined surface. In order to eliminate dust residues on the inclined surfaces, squeeze expulsion needs to be minimized during the avalanche influence. One of the methods that lower squeeze expulsion influence is to create mechanical anchoring of small dust particles in the dust layer. Since the dust particles have various shapes and sizes, grafting the dust particle surfaces can modify the surface texture, which creates mechanical interlocking (anchoring) of near neighbourhood particles. Hence, chemical modification of particle surfaces towards generating the texture characteristics, which lowers the anchoring of particles, becomes essential to minimize the dust residues on the inclined surfaces^20^. Although chemical treatment of dust particles was studied previously^20^, the acceleration of dust mitigation rate via avalanche influence is left for future study. In addition, the avalanche influence on untreated dust mitigation was studied previously^2^, the scope of the study was limited with untreated dust mitigation from inclined glass surfaces. The influence of the avalanche effect on the mitigation of solution cured dust particles is left for future study. The solution treatment changes dust surface morphology, which modifies dust particle adhesion on the glass surface. Hence, investigation of the coupling effect of adhesion and avalanche forces on the mitigation of the solution curved dust becomes essential. In the present study, dust surfaces are modified chemically using a diluted hydrofluoric acid solution. Surface morphology of treated and untreated dust is evaluated via scanning electron images while surface energies of dust are evaluated incorporating the droplet method^21^. The work of adhesion of treated and untreated dust on glass surfaces is evaluated using a micro-tribometer. A fixture is designed and built to assess the solution treated dust mitigation from inclined glass surfaces (at different tilt angles) and findings are compared with those of untreated dust under the same conditions. Dust mitigation efficiency is introduced evaluating the amount of dust mitigated from inclined surfaces at different tilting angles. Dust mitigation velocity of treated and untreated dust are monitored and evaluated using the high-speed tracking system.

## Experimental

**Figure 1:**
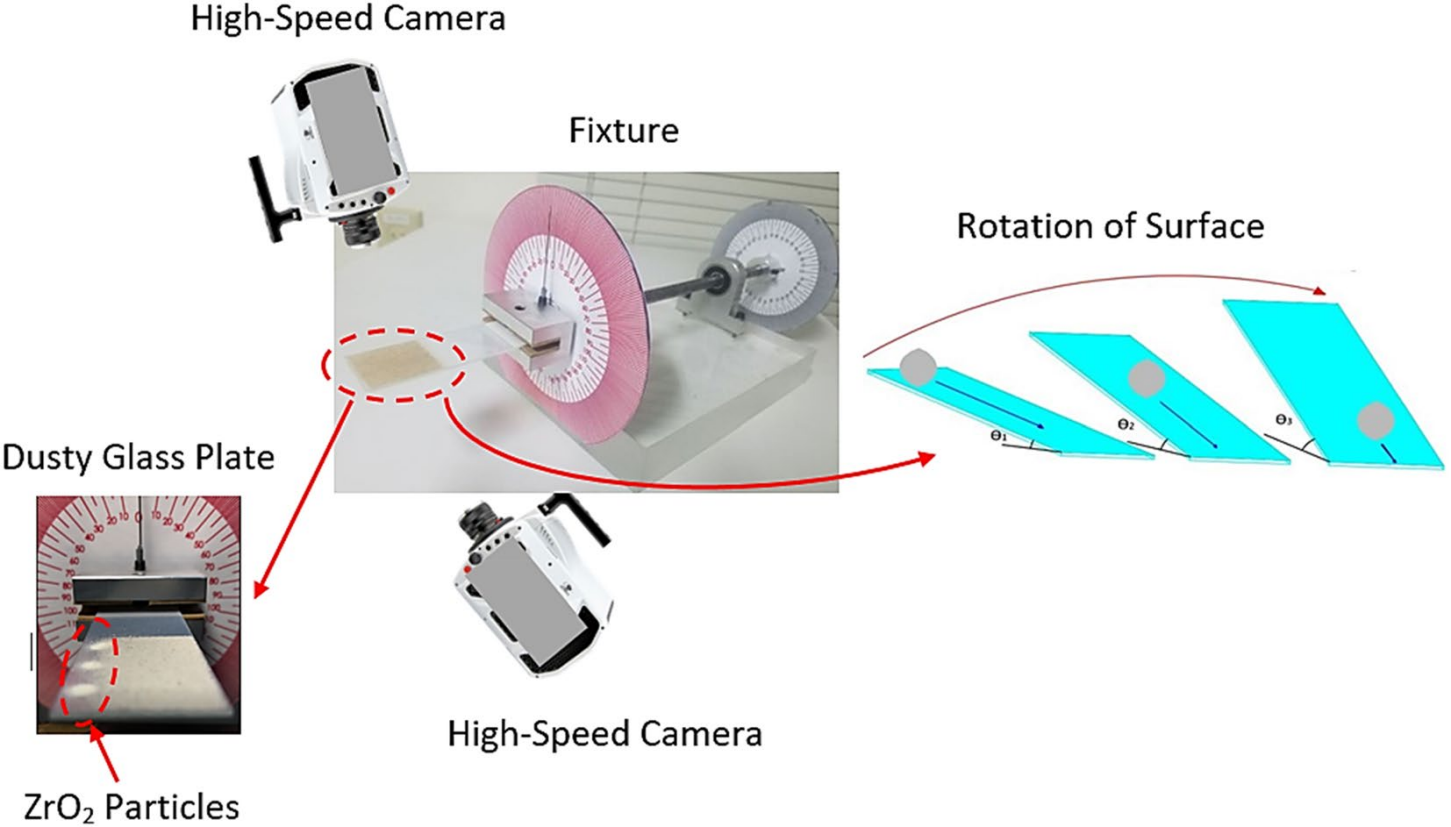
Schematic image of experimental arrangement.

Glass plates with 40 × 60 × 1 mm^3^ (width × length × thickness) were prepared and used as the samples. A fixture was designed and built to rotate the glass samples along the horizontal line, which allowed the tilting of the samples at 0.1° resolution. Figure 1 shows a schematic view of the experimental arrangements and the fixture. Environmental dust was collected from flat panel surfaces located in the Dhahran area, Saudi Arabia, by using soft brushes. Collected dust samples were characterized in terms of size, shape, and elemental constitutes using scanning electron and optical microscopes (SEM, JEOL 6460), energy dispersive spectroscopy (EDS, JEOL 6460), and X-ray diffraction (Bruker D8). Some portions of collected dust were solution treated; in which case, the solution was prepared from hydrofluoric acid at various volume concentrations (hydrofluoric acid and water mixture) within the range of 5–30% (by volume ratio of hydrofluoric acid over water). Initial tests were conducted incorporating the mixture of collect dust with the prepared solution at different concentrations. The mixture was magnetically stirred for 30 min and, later, dust particles were removed and dried at atmospheric ambient (300 K and 101.32 kPa) Later, the solution treated (cured) dust particles were examined analytically to evaluate morphological and structural changes, chemical composition, and density variations. The concentration of 20% of diluted hydrofluoric acid solution was found to be sufficient to alter the morphological, chemical, and mechanical properties of the dust particles, i.e. dust particles surface texture with sub-micron pores and pillars, and formation of fluorine compounds were observed. Hence, this concentration was selected to treat the remaining of the collected dust particles. Further analysis was conducted to assess the compounds formed on the dust particle surfaces; therefore, X-ray photoelectron spectroscopy (Thermo Scientific, Escalab 250Xi) was utilized for the solution cured dust composition analysis. In XPS tests, X-ray source of Al Kα (1486.6 eV) was utilized and the operational conditions were set at a resolution of 0.5 eV with 650 µm X-ray beam and pass energy of 100 eV for the survey scan, and 30 eV for the high resolution elemental analysis. In addition, the depth profile for XPS analysis was carried out via etching solution cured dust surfaces by Argon ion gun. Binding energies pertinent to XPS spectra were calibrated via adopting C 1 s to 284.8 eV. The pellets were formed from the solution cured dust particles and Fourier transforms infrared spectroscopy (FTIR, Thermo Fisher Scientific, Nicolet iS50) was performed.

To evaluate dust mitigation from inclined glass surfaces, a high-speed recording system (Speed Sense 9040) was used. The system was operated at 5000 frames-per-second (fps) having the megapixel resolution of 1280 × 800 and pixel size of 14 µm × 14 µm. The recording tests for dust mitigation from inclined glass surfaces were repeated 9 times assuring the repeatability of recorded data. The tracker program was used to extract the particle velocities and positions on the inclined glass surface from the high-speed data. Based on the repeatability of the data, the error was estimated at 3%. The uncertainty analysis was also conducted to determine the measurement uncertainty. Once 96% confidence-level was achieved from the data repeatability, the standard deviation of the data was evaluated via adopting the Gaussian error distribution curve, which resulted in 2% of deviation. The measurement standard uncertainty (σ_u_) could be expressed as^22^:

1 $$ \sigma_{u} = \sqrt {\mathop \smallint \nolimits_{{x_{o} }}^{{x_{n} }} \left( {x - \mu_{e} } \right)^{2} p\left( x \right)dx} $$

here, µ_e_ being the mean value of x, n is the number of measured data, and p(x) represents the probability distribution function. The probability distribution function, incorporating all dust particles influencing the locations and velocity, was initially determined from the instant correlation-plane. In addition, the probability distribution function was fitted in a suitable Gaussian-function obtaining the distribution function diameter. The uncertainty was evaluated using a least-squared-Gaussian-fitting technique. The end-result was structured (normalized) with the number of pixels of those contributing to the cross-correlation-peak. The bias error of almost 0.02 pixels was perceived because of the complex distribution of the small-peaks in the probability distribution function. Consequently, the uncertainty was estimated at 3%.

## Results and discussion

Diluted hydrofluoric acid treatment of the dust particles is realized towards reducing the dust particles pinning on the glass surfaces. The mitigation of solution cured and untreated dust from inclined glass surfaces under the avalanche influence is presented. Mitigation characteristics of solution cured and untreated dust are evaluated.

### Solution curing and dust properties

**Figure 2:**
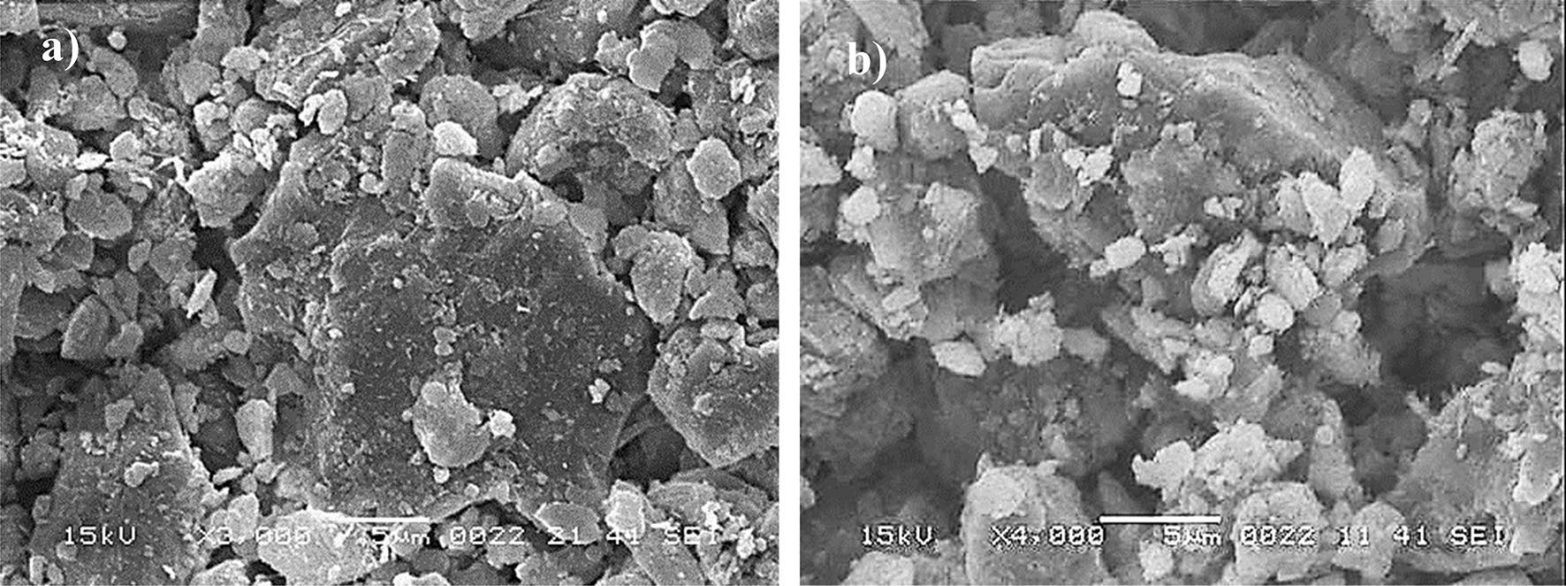
SEM images of dust particles: (**a**) dust particles with different shapes, and (**b**) small dust particles adhere on large particle surface.

**Figure 3:**
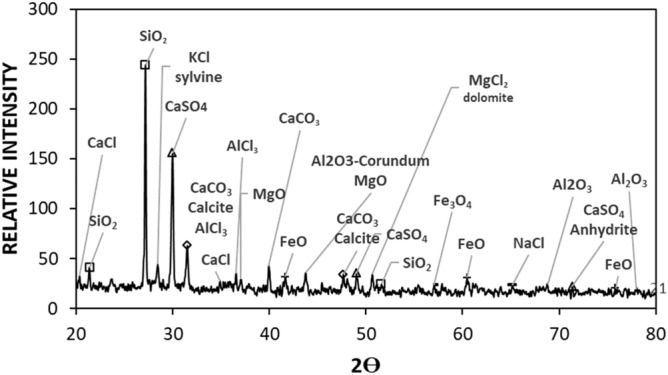
X-ray data for collected untreated dust.

**Figure 4:**
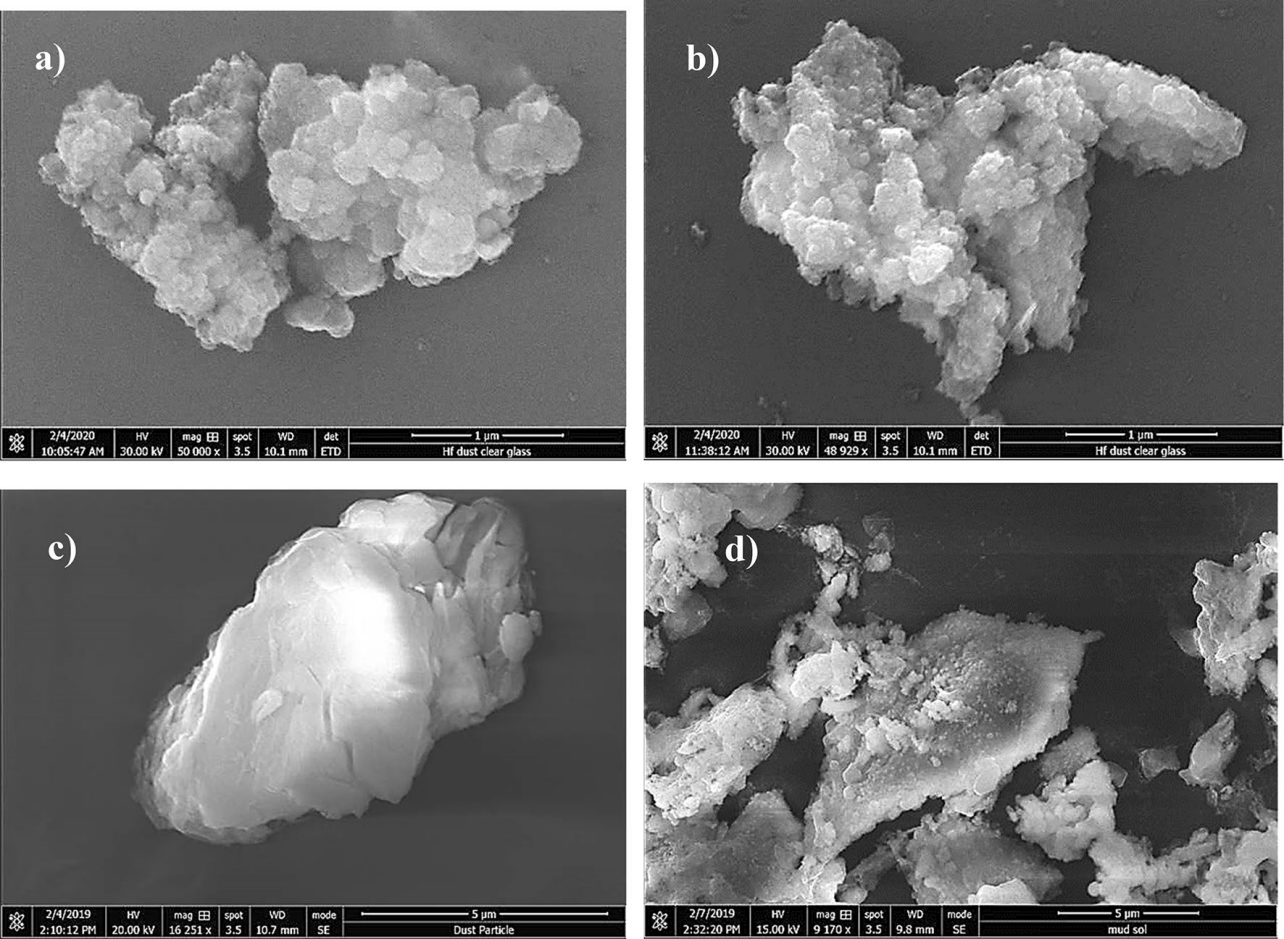
SEM images of solution cured dust particles: (**a**) grafted dust surface, (**b**) sub-micro holes on dust surface, (**c**) sub-micro crack on dust surface, and (**d**) interlocked dust particles.

SEM micrographs of untreated dust are shown in Fig. 2a,b, respectively, while Fig. 3 depicts X-ray diffraction of untreated dust. Untreated dust comprises of different sizes and shapes, and the average dust size, obtained from particle analyzer, is about 1.2 µm. Dust surface texture varies from particle to particle; nevertheless, the roughness parameter, which is determined from SEM micro-images, is in the range of 1.5–1.7. The roughness parameter is related to the ratio of the total surface ragged (pillars) area to the projected area. X-ray peaks for untreated dust reveal silica (SiO_2_), calcite (CaCO_3_), and anhydrite or gypsum (CaSO_4_) compounds on the untreated surface (Fig. 3). In addition, peaks of aggregated hematite (Fe_2_O_3_) and salt compounds (NaCl and KCl) are observed. The salt compounds in dust are probably because of local geomorphological influence since dust is collected in the Dhahran area of Saudi Arabia, which is close to the Gulf Sea. Elemental constitutes of solution treated and untreated dust is provided in Table 1. Several elements, including Fe, Si, Ca, K, Na, S, O, and Cl, are observed from Table 1 and the content of dust constitutes changes slightly as its size changes. Dust size ≤ 2 µm contains higher oxygen than the larger sizes, which may be due to prolonged suspension of small dust particles in the atmosphere^23^. Moreover, they constitute ratios of compounds formed on the dust surface that do not yield the stoichiometric ratio, i.e. values o ≠ p and s ≠ t in Na_o_Cl_p_ and K_s_Cl_t_. This creates ionic forces on the surface while small dust particles gather and form agglomerates (Fig. 2b), i.e. small particles attach on the large particle surfaces. The water solubility of these compounds is verified via pH measurements and it is noted that the pH of water dust mixture increases reaching pH = 8.6 within 10 min. Hence, salt compounds dissolve in water and change the electrolytic state of the mixture. In the case of solution cured dust, the diluted solution of hydrofluoric acid modifies the dust surface considerably. This can be observed from Fig. 4a–d, in which SEM micrographs of solution cured dust are shown. The roughness parameter of the cured dust surface varies within 1.46–1.82. Treated dust surfaces possess sub-micron holes (Fig. 4a) and fibrils like textures (Fig. 4b). The dissolution of calcite and salt compounds during the solution treatment can result in non-uniform pores of sub-micrometer sizes pillars on dust surfaces. Since salt and calcite compounds are non-uniformly distributed around the dust particle, the solution treatment gives rise to irregular texture morphology, i.e. non-uniformly distributed sub-micro pillars and pores around the dust particle surface (Fig. 4a,b). The density differences between the newly formed fluorine compounds (such as CaF_2_, 3180 kg/m^3^), and the density of untreated dust (~ 2800 kg/m^3^) can cause mechanical strain under the volume contraction/expansion. This causes some small micro-size cracks appearing on the dust particle surface (Fig. 4c). In addition, closely positioned solution treated dust particles almost mechanically interlocked to each other(Fig. 4d) because of sub-micron fibrils like textures on the dust particle surface (Fig. 4a); hence, small size particle adhesion on large dust surfaces replaces with almost mechanical interlocking. The alteration of surface morphology of the dust particles is because of the chemical reactions occurring during the diluted hydrofluoric acid treatment. The possible chemical reactions taking between the dust compounds and the diluted hydrofluoric acid may be expressed as follows:

2 $$ {\text{SiO}}_{2} + 6{\text{HF}} \to {\text{H}}_{{2{ }}} {\text{SiF}}_{6} + 2{\text{H}}_{2} {\text{O}} $$

**Table 1:** Elemental constitute of dust particles (wt%).

	Size (μm)	Si	Ca	Na	S	Mg	K	Fe	Cl	F	O
Collected	≥ 1.2	11.6	8.5	2.1	1.1	2.5	0.8	1.2	0.4	0	Bal.
Collected	< 1.2	10.4	7.7	2.5	2.1	1.8	1.2	1.1	1.1	0	Bal.
Solution treated	≥ 1.2	8.1	8.5	1.3	1.2	2.3	0.6	0.9	0.2	28	Bal.
solution treated	< 1.2	7.6	7.8	1.4	1.1	1.6	0.8	0.8	0.3	32	Bal.

It is worth to mention that average size of dust particles is 1.2 µm.

After the reaction hexafluorosilicate acid (${\text{H}}_{{2{ }}} {\text{SiF}}_{6}$) can be formed. Since, salt compounds (NaCl, KCl) are present in the untreated dust particles (Fig. 3) salt compounds can react with hexafluorosilicate and forms sodium hexafluorosilicate, i.e.:

3 $$ 2{\text{NaCl}} + {\text{H}}_{{2{ }}} {\text{SiF}}_{6} \to {\text{Na}}_{2} {\text{SiF}}_{6} { }\left( {\text{s}} \right) + 2{\text{HCl}} \left( {\text{l}} \right) $$

Similarly, for KCl, potassium hexafluorosilicate is formed, i.e.:

4 $$ 2{\text{KCl}} + {\text{H}}_{{2{ }}} {\text{SiF}}_{6} \to {\text{K}}_{2} {\text{SiF}}_{6} + 2{\text{HCl}} \left( {\text{l}} \right) $$

${\text{Na}}_{2} {\text{SiF}}_{6}$ and ${\text{K}}_{2} {\text{SiF}}_{6}$ are the crystalline solids, which can dissolve in water (solution mixture) and they precipitate and settle on the container surface because of higher density than water. However, the compound of calcite (CaCO_3_) in dust can also react with HCl (l), which is the partial product of salt and hexafluorosilicate (${\text{H}}_{{2{ }}} {\text{SiF}}_{6}$) reactions, hence:

5 $$ {\text{CaCO}}_{3} + 2{\text{HCl}} \to {\text{CaCl}}_{2} + {\text{CO}}_{2} + {\text{H}}_{2} {\text{O}} $$

Here, CaCl_2_ is highly soluble salt in water. In addition, the compound of calcite (CaCO_3_) reacts with hydrofluoric acid and forms calcium fluoride, i.e.:

6 $$ {\text{CaCO}}_{3} + 2{\text{HF}} \to {\text{CaF}}_{2} + {\text{CO}}_{2} + {\text{H}}_{2} {\text{O}} $$

Magnesium oxide (MgO) compound can also react with hydrofluoric acid and forms magnesium fluoride, i.e.:

7 $$ {\text{MgO}} + 2{\text{HF}} \to {\text{MgF}}_{2} + {\text{H}}_{2} {\text{O}} $$

**Figure 5:**
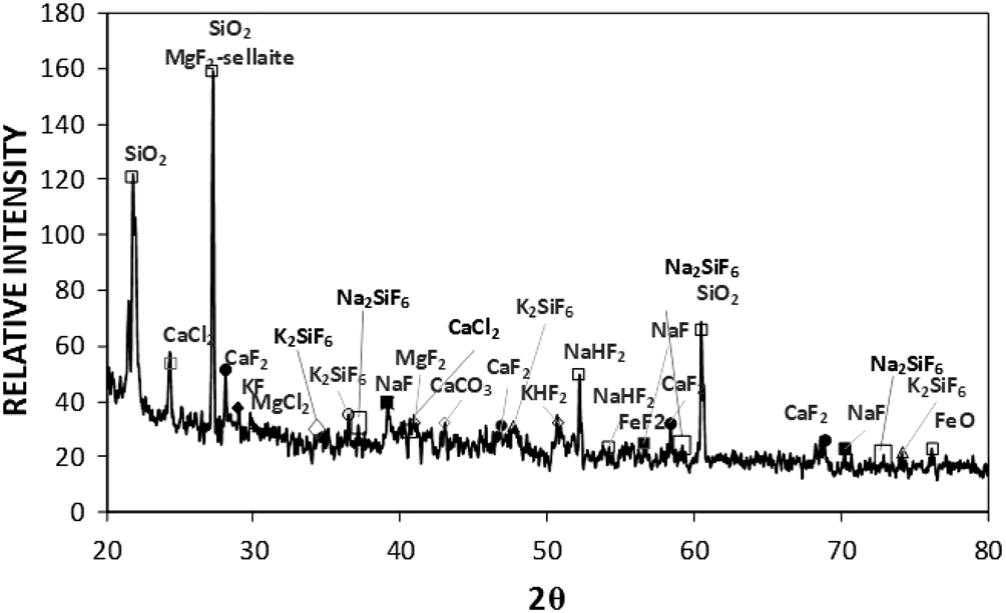
X-ray data for solution cured dust.

**Figure 6:**
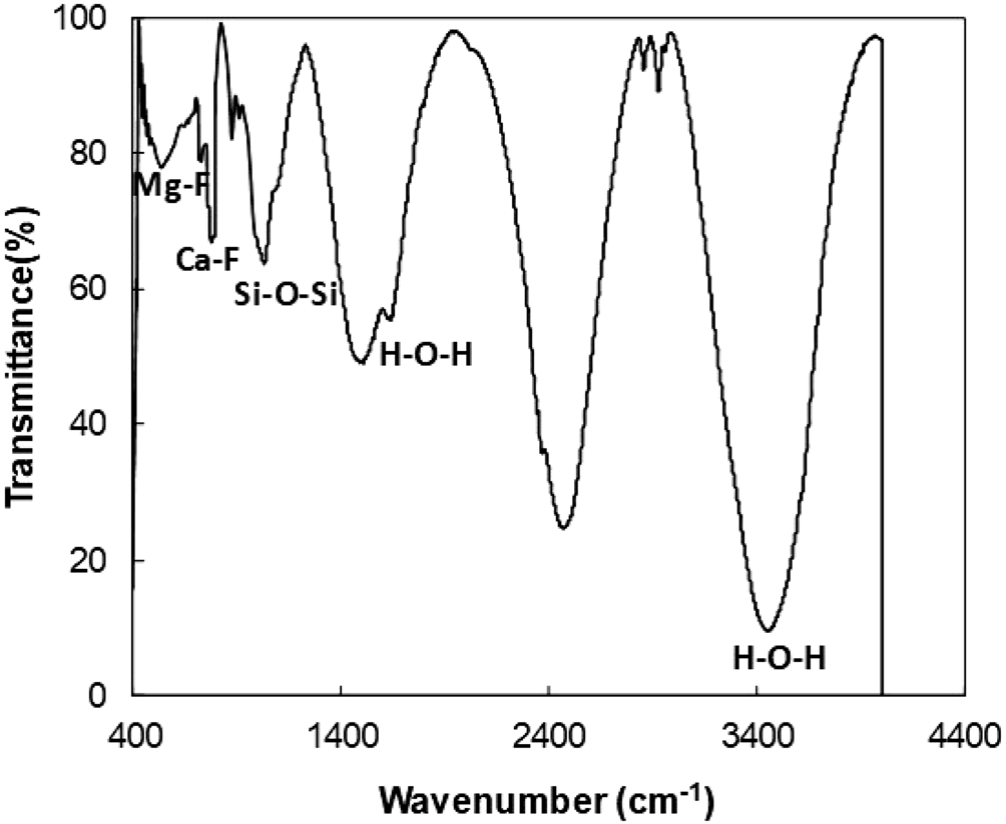
FTIR data for solution cured dust.

MgF_2_ is the fluorescent crystals, which stays on dust surfaces (non-soluble in water). Table 1 gives the elemental composition of dust after solution treatment by diluted hydrofluoric acid. The elemental constitutes of solution treated (cured) dust differ from the constituents of untreated dust (Table 1). Solution cured dust possesses the same elements of untreated dust (Table 1) and additionally, fluorine is observed while demonstrating fluorine compounds in the dust particle surfaces. Figure 5 depicts an X-ray diffraction chart of cured dust, which demonstrates various fluorine compounds such as ${\text{CaF}}_{2}$, ${\text{MgF}}_{2}$, ${\text{Na}}_{2} {\text{SiF}}_{6}$, ${\text{K}}_{2} {\text{SiF}}_{6}$. Although ${\text{Na}}_{2} {\text{SiF}}_{6}$ and ${\text{K}}_{2} {\text{SiF}}_{6}$ salts dissolve in water, X-ray diffraction chart shows that some of these compounds remain on dust surfaces. Since, ${\text{CaF}}_{2}$ and ${\text{MgF}}_{2}$ do not dissolve in solution during curing, they remain as a compound on dust surfaces. Moreover, CaCO_3_, SiO_2_, and FeO peaks are also observed for the solution cured dust. These compounds are the remaining of the untreated dust, most probably just below the surface skin. Moreover, calcite (CaCO_3_) appears as rhombohedral form in solution cured dust; in which case, 2θ is shifted to 43.1° (PDF card Nr. 24–007-calcite-CaCO_3_-Rhombohedral) in the XRD-diffractogram (Fig. 5). It is worth to note that CuKα radiation source is used in X-ray diffraction analysis, which has well above micrometer penetration depth in CaCO_3_^24^. For further evaluation of cured dust characteristics X-ray photoelectron spectroscopy (XPS) is conducted. XPS data for cured dust (Table 2) demonstrate increased calcium and fluorine and diminishing oxygen and silicon contents. The Fourier infrared spectroscopy (FTIR) data for cured dust are shown in Fig. 6. The peak at1128 cm^-1^ represents Si–O-Si rotational vibration^25^; hence, it is probable that polysiloxane is formed via ion release from Na_2_SiF_6_ on cured dust surface^26^. The Mg-F peak is observed at 477 cm^−1^ because of stretching vibration^27^. The peaks at 3448 cm^−1^ and 1661 cm^−1^ are typical bending vibration behavior of hydroxyl groups (H–O–H)^28^. Moreover, Ca-F stretching vibration peak occurs at 775 cm^−1^^29^.

**Table 2:** XPS data for untreated and solution cured dusts.

	O1s	C1s	Si2p	Ca2p	N1s	Al2p	F1s
**Prior etching**							
Collected	52.11	23.19	10.82	5.17	0.27	5.71	1.10
Solution treated	30.19	21.93	3.82	10.04	1.41	2.61	29.99
**After 20 s etching**							
Collected	58.26	9.15	12.75	7.14	1.03	7.42	0.00
Solution treated	26.86	8.72	7.60	11.99	0.00	3.42	41.32

**Figure 7:**
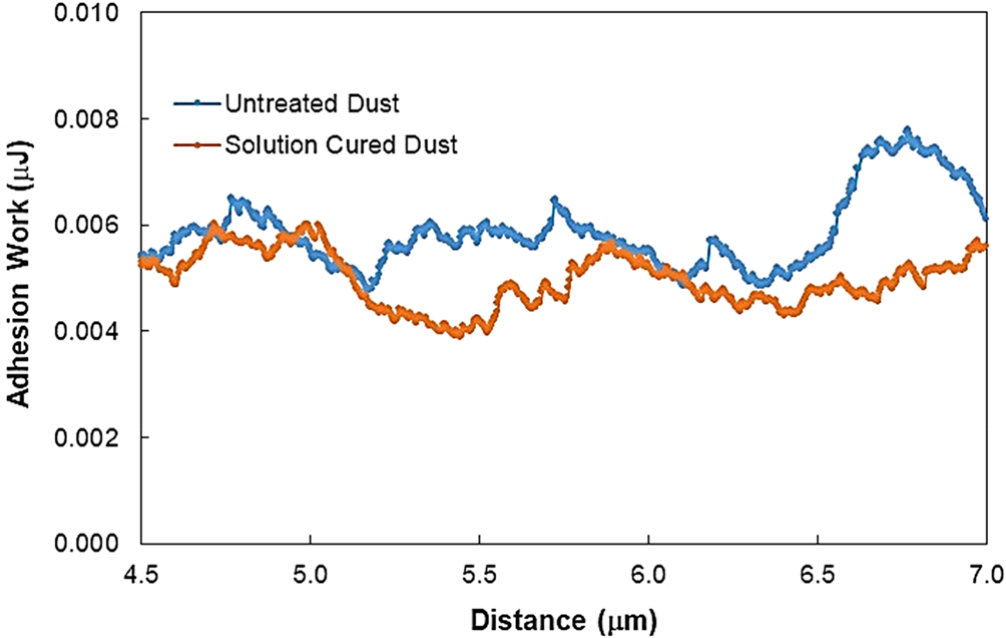
Work adhesion along the scanning (scratching) length on dusty glass surface.

The surface free energy of the solution cured dust is determined adopting the liquid droplet method^30,31^. Water, glycerol, and ethylene glycol are accommodated in the measurements. Since the dust particles are loose like-powders, solution cured dust pellets are formed initially and the selected liquid contact angle measurements are carried out on the pellet surface. In addition, the method developed by Washburn^32^ is also adopted to measure the contact angles of the liquids. The contact angle measurements are realized while using the method described in the early work^33^. The Washburn method relies on the rate of liquid draw-up by the particles. Hence, solution cured dust particles are filled into a 3 mm diameter glass tube, which is placed on a small liquid holder. The solution cured dust particles draw-up the liquid from liquid holder through an infusion. The mass increase per unit time in the tube under liquid draw-up can be expressed by the Washburn equation, i.e.: $\frac{{\left( {\Delta m} \right)^{2} }}{\Delta t} = \frac{{c.\rho^{2} \gamma cos\theta }}{\mu }$, here Δm being mass gain, Δt represents mass gain (flow-time) duration, c is the capillary constant for dust, ρ is liquid density, θ being liquid contact angle, µ corresponds to liquid viscosity. The value of c (capillary constant) is determined via placing n-hexane in the liquid holder. This results in zero contact angle (θ = 0). Therefore, the value of c is obtained as 5.52 ± 1.3 × 10^–16^ m^−5^ for the solution cured dust particles. Several tests are carried out and the experimental error is estimated as 8%. The attainment of slightly large measurement error is because of each refilling of a glass tube, the dust particles are not same in terms of sizes and shapes, which effects the test results. Nevertheless, the experimental errors are less than 10% and similar arguments for the measurements are also reported in the early study^34^. The water contact angle measured from the Washburn method is 38.4° ± 3° while the contact angle measured results in 38.7° ± 3° for water. Hence, both methods result in very close values of the water contact angle. The contact angle measurements are repeated for the other selected liquids for the free surface energy assessment of the solution cured dust. The surface free energy can be obtained through: $\gamma_{L} \left( {\cos \theta + 1} \right) = 2\sqrt {\gamma_{S}^{L} .\gamma_{L}^{L} } + 2\sqrt {\gamma_{S}^{ + } .\gamma_{L}^{ - } } + 2\sqrt {\gamma_{S}^{ - } .\gamma_{L}^{ + } }$^30,31,35^. Here, L and S being the liquid and solid phases, γ_*S*_ represents the surface energy for solid, γ_*S-L*_ is the tension at the solid–liquid interface, γ_*L*_ being the liquid surface tension, θ is contact angle, γ + and γ- are electron acceptor and donor parameters. Table 3 provides Lifshitz-van der Walls components and electron-donor parameters^30,31,35^. Moreover, the surface energy for solution cured dust is found to be about 94.8 ± 6.2 mJ/m^2^. However, the surface free energy for untreated dust is 112.3 ± 7.8 mJ/m^2^. Hence, solution treatment reduces the surface free energy of the dust particles. This is because of the fluorine compounds formed on the solution cured dust particles^36^. The adhesion of the dust particles on glass surface is important for removal from surface. The adhesion force formulation between the different surfaces is presented previously^37^. Moreover, the formulation presented earlier^38^ is adopted to account for the roughness of the dust particles; hence, the force of adhesion of the particle ($F_{Ad}$) can be written as: $F_{Ad} = \frac{{AR_{pd} }}{{12Z_{0}^{2} }}\left( {\frac{1}{{1 + \frac{{R_{pd} }}{{1.48r_{s} }}}} + \frac{1}{{\left( {1 + \frac{{1.48r_{s} }}{{Z_{0} }}} \right)^{2} }}} \right)$, here *A* being the Hamaker constant, which is A = 0.48 × 10^–20^ J for SiO_2_ (main constitute of a dust particle)^39^, *R*_*pd*_ represents the equivalent radius (hydraulic radius) of the particle, and *Z*_*o*_ corresponds to the particle spacing from the surface, $\varepsilon$ is roughness of the sample (glass) surface, and *r*_*s*_ is the dust particle surface roughness parameter. The sample (glass) roughness is considered as in the order of 50 nm and the roughness parameter for the dust particle is evaluated by SEM images and it is estimated as *r*_*s*_ ≅ 40 nm. Hence, the adhesion force estimated from the Rabinoich formulation^38^ is about 1.7 × 10^–12^ N for 10 µm dust particle. To verify the dust particle adhesion on the sample surface, the adhesion force measurement by the atomic force microscopy (AFM) is carried out. In the friction mode, the AFM probe deflection and the probe voltage provide the relation for the force of adhesion, which is: $F = k\sigma_{d} \Delta V$, where *k* being AFM probe spring constant (N/m), *σ*_*d*_ represents the slope of deflection (*Δz*/*ΔV*, m/V) and *ΔV* corresponds to the recorded probe voltage (mV) for the deflection^40^. The AFM probe is silicon nitride and it has $k\sigma_{d}$ = 5.80275 × 10^–15^ N/mV characteristic. The AFM probe voltage measured for 10 μm dust particle on the glass surface is 350 mV, which results in ~ 2.03 × 10^–12^ N for the adhesion force of 10 μm dust particle on the sample surface. As comparing the adhesion force predicted from Rabinoich formulation (1.7 × 10^–12^ N for) and AFM measurement, both results are considerably close. The small variation in adhesion force can be related to the particle roughness parameter and the particle roundness which differ from particle to particle, yet, the average size of the particle being same. Nevertheless, the findings are in the same order. Since the small dust particles attach on the large size dust particle (Fig. 2b) the particle adhesion causes the particle clustering. The adhesion between the small and large dust particles is formulated previously, which results the particle adhesion force ($F_{Pad}$) in the form of: $F_{Pad} = \frac{{AR_{s} }}{{6Z_{o} }}$^14^, where *R*_*s*_ is reduced dust radius, which is: $R_{s} = \frac{{s_{1} .s_{2} }}{{s_{1} + s_{2} }}$, where *s*_*1*_ and *s*_*2*_ are the hydraulic radius of each particle in contact (adhere together). Introducing, dust particles having the hydraulic radius of 0.3 μm (small size) and 5 μm (large particle), and spacing between them (*Z*_*o*_) is 50 nm, the *F*_*Pad*_ yields 4.48 × 10^–12^ N, which is larger than the 5 μm radius dust particle adhesion on the glass surface (2.03 × 10^–12^ N). Hence, the small size dust particles adheres strongly on to the large size dust particle surface. Moreover, the work of adhesion of untreated and cured dust on the glass surface is measured using the micro-tribometer (CSM Instruments, Micro Scratch Tester). The work of adhesion is determined from data for the tangential force, which is required to remove of particles from the glass surface. Hence, tangential force variation along the length on the glass surface is obtained, i.e. tangential force (*F*_*tan*_) is measured along the probe path L, which is the total dust removal length along the scanning direction. Later, the frictional force (*F*_*f*_) generated on the plain glass surface is subtracted from *F*_*tan*_, i.e. $F_{add} = F_{tan} - F_{f} { }$, here Fadd is the adhesion force required to remove dust from the plain glass surface. It is worth to note that the frictional force on the glass surface is measured, by the micro-tribometer, without presence of dust on the surface. The work of adhesion ($W_{ad}$) along L is obtained via integration of adhesion force over the scanning length (L), i.e.: $W_{ad} = \int_{0}^{L} {F_{ad} } dl$. Figure 7 depicts work of adhesion along micro-tribometer probe path (L) for untreated and solution cured dusts on the glass surface. The maximum micro-tribometer probe path (L) extends L_d_, which is the maximum scanning path utilized in the measurements. Since the untreated and solution cured dust particles have various shapes and sizes, the contact area of individual dust particles on the glass surface varies; hence, the interfacial forces between each dust particle and the surface differs. This gives rise to irregular variation of the work of adhesion along the probe path. Nevertheless, the work adhesion is smaller for the solution cured dust than the untreated dust. This is because of the reduced interfacial forces between the cured dust particles and the glass surface, i.e. formation of fluorine compounds on dust surface lowers interfacial forces for pinning. In addition, modification of surface morphology of the dust particles (Fig. 4a) after solution curing reduces the contact area between the solution cured particles and the glass surface. This also contributes to reduced tangential force requiring to remove dust from the glass surface. The overall work of adhesion (W_ad_) can be determined through $W_{ad} = \int_{0}^{{L_{d} }} {F_{add} } dl$. The overall work of adhesion is 0.94 µJ for untreated dust while it is 0.56 µJ for solution cured dust on the glass surface. Hence, solution curing of dust particles results in almost 40% less adhesion than the untreated dust on the glass surface. It is worth to mention that the clustering of dust particles contribute to the work of adhesion, since the force of adhesion among the clustered particles is larger than the dust particle adhesion on the glass surface. Hence, treatment of dust particles reduces the surface free energy, which in turn lowers the adhesion among the clustered dust particles while lowering the work of adhesion.

**Table 3:** Lifshitz-van der Walls components and electron-donor parameters used in the simulation^29,30,34^.

	$\gamma_{L}$ (mJ/m^2^)	$\gamma_{L}^{L}$ (mJ/m^2^)	$\gamma_{L}^{ + }$ (mJ/m^2^)	$\gamma_{L}^{ - }$ (mJ/m^2^)
Water	72.8	21.80	25.5	25.5
Glycerol	63.3	33.11	10.74	21.23
Ethylene glycol	48.2	31.09	6.59	11.16

### Dust removal under the influence of avalanche

**Figure 8:**
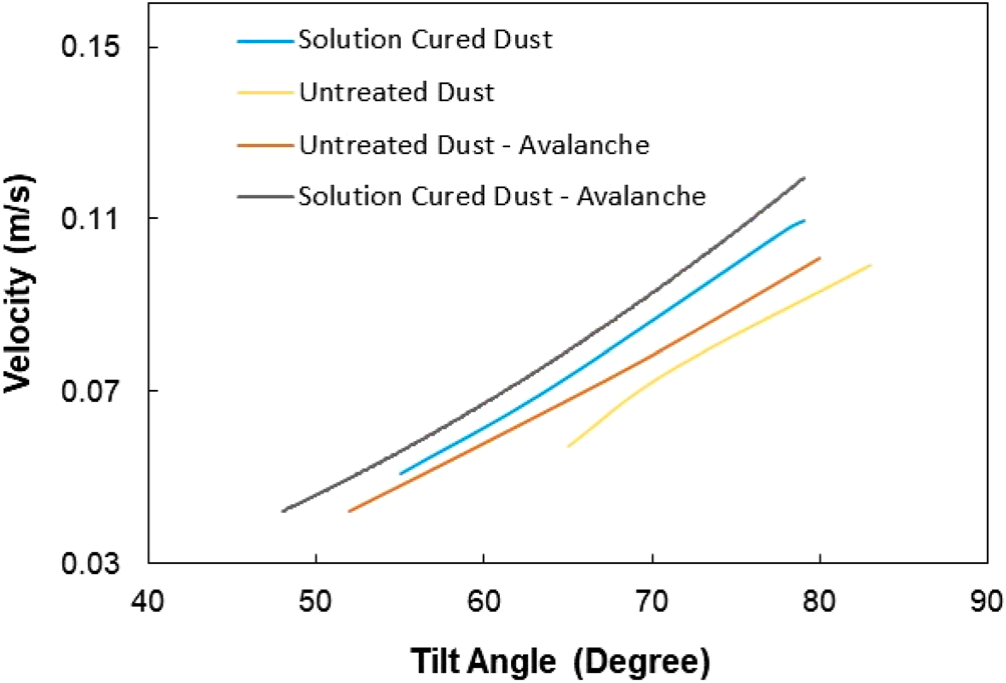
Average dust particle velocity with glass surface tilting angle for solution cured and untreated dusts with and without avalanche influence.

**Figure 9:**
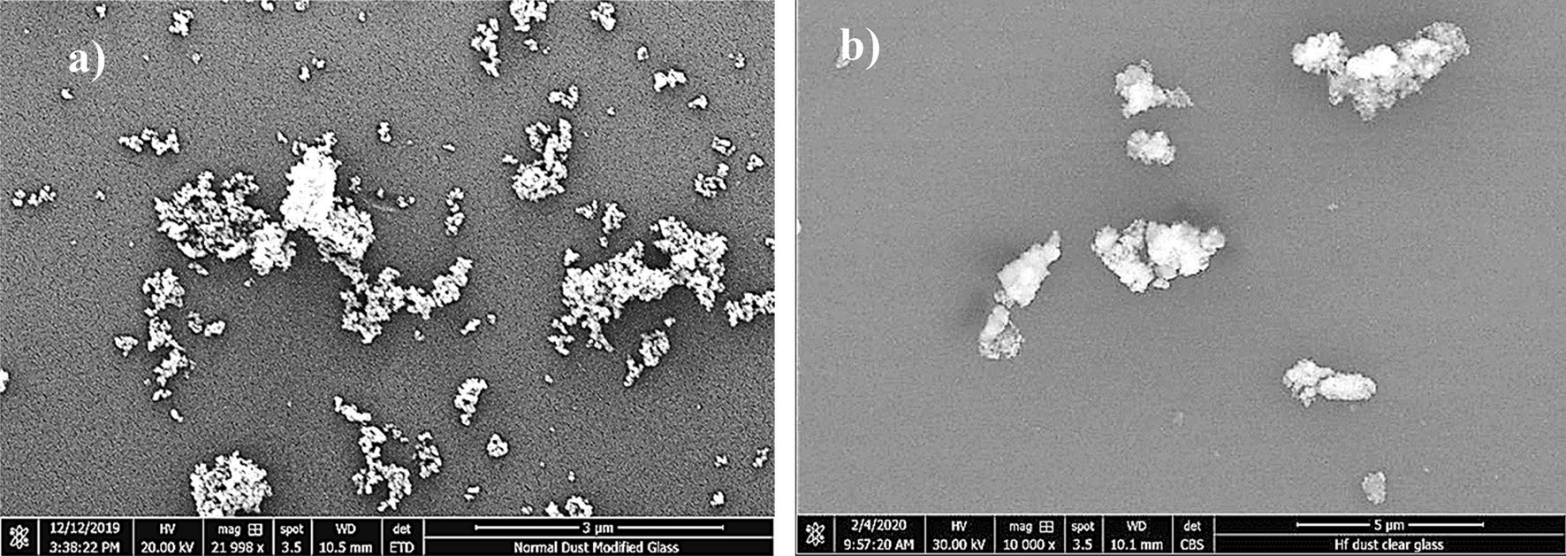
Dust residues on glass surface: (**a**) untreated dust, and (**b**) solution cured dust.

**Figure 10:**
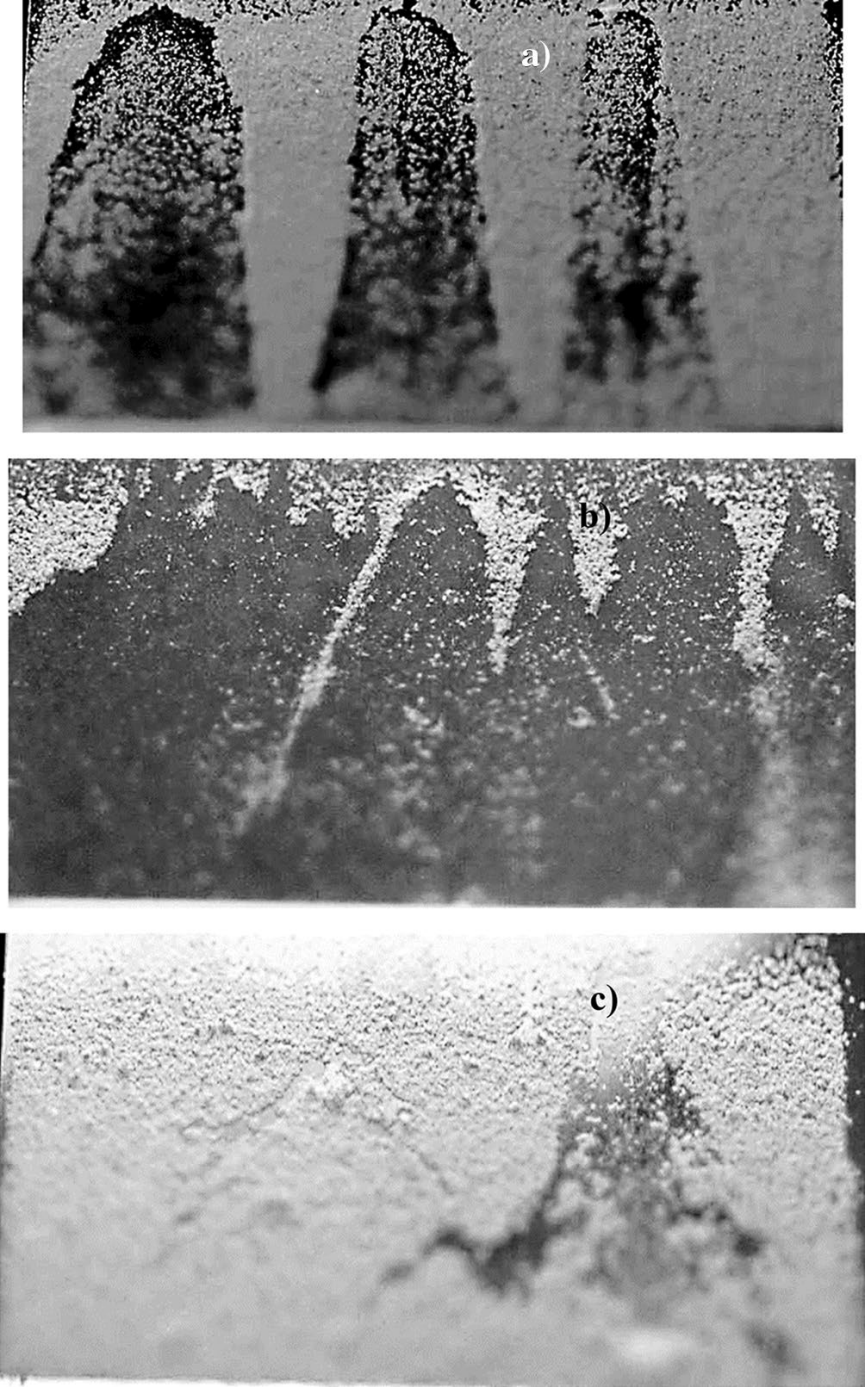
High-speed camera images of mitigated dust from surfaces: (**a**) untreated dust with avalanche influence, (**b**) solution cured dust with avalanche influence, and (**c**) untreated dust without avalanche influence. Inclination angle of the surface treated and untreated dust with avalanche influence is 57° while untreated dust without avalanche influence is 65°.

**Figure 11:**
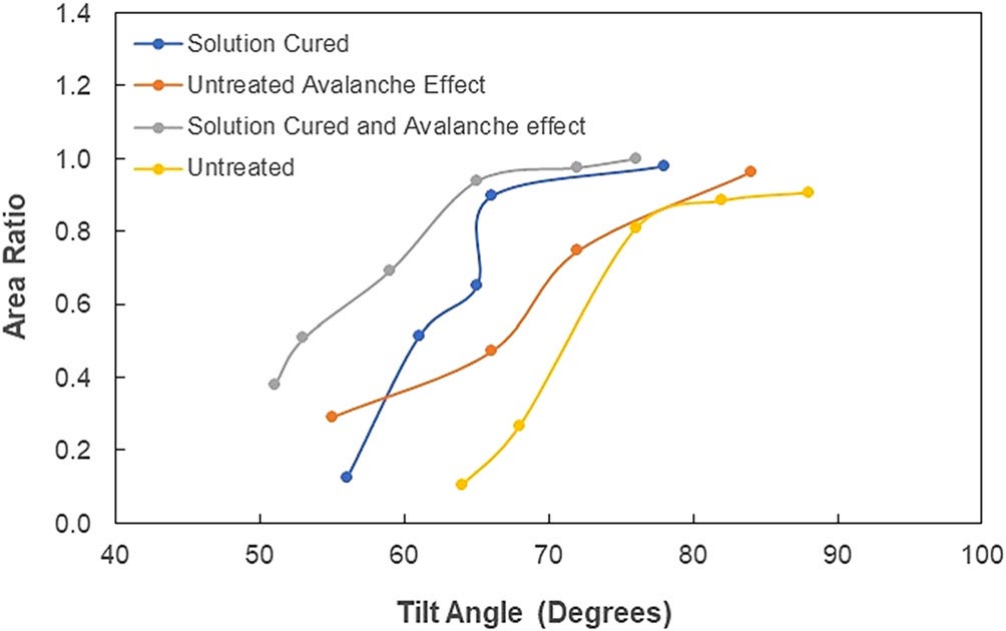
Area ratio (*A*_*clean*_/*A*_*Total*_, *A*_*clean*_ is area of dust mitigated surface and *A*_*Total*_ is area of dusty glass surface) with glass surface tilting angle for solution cured and untreated dusts with and without avalanche influence.

**Figure 12:**
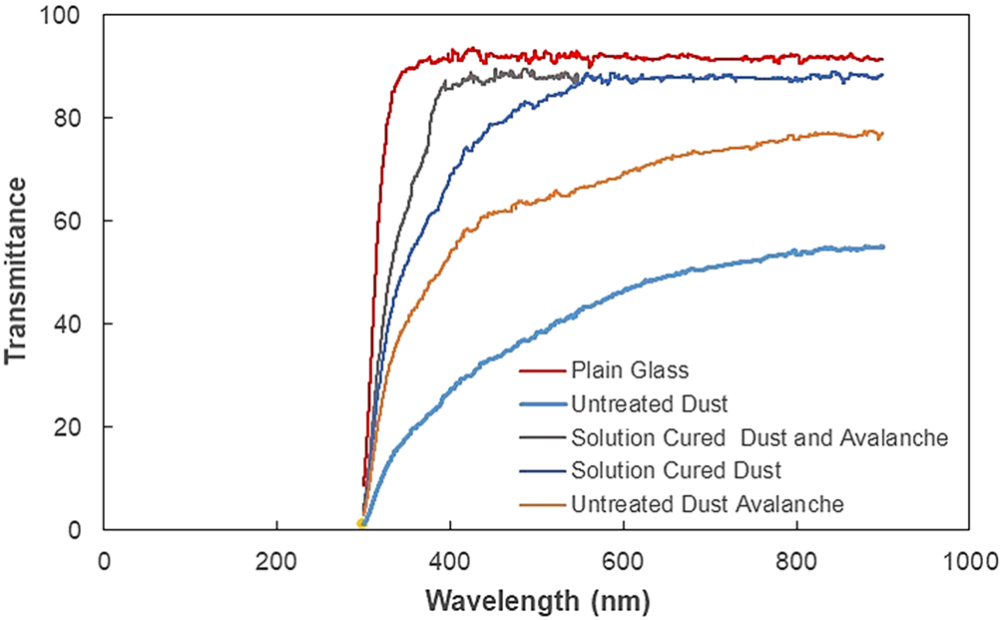
Optical transmittance of dust mitigated glass surfaces.

In order to evaluate the influence of the tilt angle of the glass surface on the initiation of dust removal under the influence of avalanche, the tests are conducted and initiation of dust mitigation and dust particle velocities are monitored by the high-speed recording system. The tracker software is, later, used to analyze the recorded data in terms of the positions of particles and their velocities on the inclined glass surface. In addition, the velocity data obtained for solution cured dust and untreated dust without avalanche influence are also provided for comparison. Figure 8 shows the average velocity of the dust particles with the tilt angle of the surface for avalanche and none-avalanche arrangements. It is worth to mention that average velocity of the dust particles are obtained from the high speed recorded data (high-speed camera data), which are analyzed using the tracker program. Initiation of dust mitigation occurs at a certain tilt angle of the glass surface and the avalanche influence gives rise to the departure of dust particles at low inclination angles of the glass surface. Hence, introducing ZrO_2_ particles creates the avalanche effect and causes the early departure of the dust particles mitigating from the glass surface, which is more apparent for the solution cured dust. The work adhesion of the solution cured dust particles on the glass surface is almost 40% less than those of untreated dust; hence, for solution cured dust, the gravitational force becomes larger than the pinning force at small tilt angles of the glass surface. The avalanche influence on both solutions cured and untreated dust particles are notable as compared to the cases without the avalanche effect in Fig. 8. However, the mechanical interlocking of solution cured dust particles gives rise to mass dust motion on the inclined surface. This enhances the velocity of the solution cured dust and the mitigation velocity becomes larger for solution cured dust than that of the untreated dust. Moreover, ZrO_2_ particles have an average size of 50 µm, which differs significantly from the average size of the dust particles (1.2 µm). Although the size effect of avalanching particles causes segregation of large and small particles via kinetic sieving^18^ and squeeze expulsion^19^, this influence is not observed from the high speed-camera data. This is because of one or all of the followings: (1) mitigation velocity of the dust particles are low at which the kinetic sieving becomes small^18^, (2) clustering of mitigating dust particles, due to mechanical anchoring, causes the large size of clustered dust particles, which becomes comparable to ZrO_2_ particle size. Moreover, the segregation of dust particles due to particle size effect is also not observed for untreated dust particles. This is most likely that the untreated dust particles cluster during the mitigation, i.e. small dust particles attach on large dust particle surfaces and form cluster-like structures (Fig. 2b). However, some small dust particles remain on the glass surface because of strong adhesion between the dust particles and the glass surface. It is worth to mention that small dust particles possess large ionic forces, which cause strong adhesion on surfaces. This can be observed in Fig. 9a, in which SEM micrographs of dust remains (residues) on the glass surface are shown after dust mitigation. Also, SEM micrographs of solution cured dust residues on the glass surface after dust mitigation is also shown in Fig. 9b. In general, dust residues are small and scattered over the surface, which is more apparent for the solution cured dust case. However, untreated dust residues demonstrate the clustered-like structures on the glass surface. The energy-dispersive spectroscopic analysis shows that untreated dust residues possess the salt compounds with non-stoichiometric elemental ratios. Hence, the ionic forces developed on the dust particles contribute to the adhesion of these particles on the glass surface. Nevertheless, dust residues do not cover the large area on the glass surface. In the case of solution cured dust residues, the size of the particles is small and energy dispersive spectroscopic analysis revealed that silicon content is high while no fluorine is observed in the cured dust residues. Therefore, few dust residues are not properly solution treated, which maybe because of the low concentration of diluted hydrofluoric acid treatment. To evaluate the area of the glass surface cleaned (dust mitigated) for untreated and solution treated dust cases, the ratio of dust mitigated surface area over the total surface area of the dusty glass surface is extracted from the high-speed camera images. Figure 10 shows optical images extracted from high-speed records for untreated and solution cured dust cases at the same inclination angle under the avalanche influence. Also, the image of untreated dust particles without avalanche influence at the same inclination angle is provided in Fig. 10 for comparison. The solution cured dust particles are mitigated in bulks under the avalanche influence and the coverage of the dust mitigated area remains significantly larger than that of the untreated dust under the avalanche influence. Hence, the interlocking of the solution cured dust particles to pull down the neighboring particles while causing clustered particles to mitigate from the surface. Contrarily, the mitigation of the untreated dust particles occurs along the ZrO_2_ path; however, dust adhesion between each other under ionic forces enables some untreated dust particles to mitigate along the path of the ZrO_2_ particles. It is worth noting that as untreated dust particles slide down on the glass surface, their velocity increases and the force of inertia enhances the amount of dust particles mitigating in the neighbourhood path of the ZrO_2_ particles, particularly in the region towards the glass end. This creates an expanding dust mitigated area on the glass surface while forming a trapezium shape on the surface (Fig. 10). Besides, the reduced adhesion force between the solution cured dust particles and the glass surface contributes to a large area of the mitigated particles on the glass surface. For dust particles without avalanche effect (Fig. 10), the mitigated dust particles demonstrate irregular paths on the glass surface, and the area corresponding to dust mitigation appears to be arbitrary shapes unlike that of the solution cured dust particles. Since untreated dust results in large work of adhesion, the untreated dust particles strongly adhere to the glass surface. Some large dust particles, such as ~ 20 µm, with reduced interfacial contact area (due to high roughness of the dust surface) on the glass surface, give rise to an early departure from the inclined glass surface, i.e. these particles can act as mitigation centers in the dusty layer. Hence, they create a local avalanche influence on mitigating small size dust particles on the glass surface. Since these particles are randomly distributed on the surface, the area covered by the mitigated dust particles becomes randomly shaped on the glass surface. Figure 11 shows the variation of the ratio of dust mitigated area over the dusty glass surface area with the inclination angle of the surface for solution cured and untreated dust particles under the avalanche influence. Also, the area ratio for untreated dust without the avalanche effect is included in Fig. 11 for comparison. For the case of avalanche influence, area ratio starts increasing at low inclination angles of the surface and the area ratio becomes almost unity at smaller inclination angles as compared to the case at which none-avalanche influence is present. Hence, the external power required tilting the glass surface becomes less as the avalanche influence is introduced. To determine the optical properties of the dust mitigated glass surfaces, UV optical transmittance of the surfaces is evaluated for each dust mitigation case. Figure 12 depicts the optical transmittance of the dust mitigated and dusty glass surfaces, and as received glass surface is also included for comparison. The optical transmittance reduces considerably as the surface is covered by the dust particles of about 50 µm. Dust mitigation under the gravitational influence improves the optical transmittance of the glass surface considerably. The difference between the optical transmittance of glass surfaces due to solution cured dust mitigation under the avalanche influence and the clean glass surface is almost 1%. This difference is about 15% for solution cured dust without avalanche influence up to 450 nm wavelength. However, the optical transmittance difference becomes almost 35% for untreated dust mitigation without the avalanche effect. Although dust mitigation from the tilted glass surface improves the optical transmittance, this improvement reaches the maximum for the solution cured dust particles under the avalanche influence. Hence, a combination of solution curing of dust particles and avalanche influence cleans almost the entire glass surface while providing the maximum optical transmittance.

## Conclusion

Accelerated dust mitigation from the glass surfaces under the avalanche influence is considered. Dust collected from PV panel surfaces in the local area of Dhahran, in Saudi Arabia, are treated using the diluted solution of low concentrated hydrofluoric acid. Solution treatment of the dust particles increases the particle roughness parameter from 1.46 to 1.82 and causes fluorine compounds forming (CaF_2_, MgF_2_, ${\text{Na}}_{2} {\text{SiF}}_{6}$ and ${\text{K}}_{2} {\text{SiF}}_{6}$) on the particle surfaces through the chemical reactions. The avalanche influence is created on the untreated and solution cured dust particles on the inclined glass surface. Since the gravitational potential is utilized generating the avalanche influence, the large particles (~ 50 µm) with higher density (5200 kg/m^3^) than the dust particles (1.2 µm average size and 2800 kg/m^3^) are located on the top surface of the glass samples. The solution treated and untreated dust mitigations on the inclined glass surface are monitored using the high-speed recording facility. The work adhesion between the dust particles and the glass surface is evaluated incorporating the tangential and frictional forces, which are obtained from the micro-tribometer data. Findings reveal that the solution cured dust particles result in almost 40% less work of adhesion as compared to that of the untreated dust particles. Hence, dust pinning on the glass surface reduces as the dust particles are cured in the low concentration hydrofluoric acid solution. The untreated dust particles possess various compounds and some salt compounds have a non-stoichiometric elemental ratio while creating additional ionic forces particularly for small size dust particles. SEM micrographs of these particles reveal that small size dust particles attach on the surface of the large size dust particles. The solution treatment adopted eliminates the adhesion of the small dust particles on to the large particles and changes the dust particle morphology while creating sub-micron size pores and needle-like pillars on the particle surfaces. The change of dust surface texture lowers the contact area among the solution cured dust particles; however, it enhances the mechanical interlocking of dust particles in close neighbourhoods. Hence, low adhesion of the solution cured dust particles results in mitigation on the glass surface at low inclination angles. The avalanche effect introduced accelerates the mitigation of solution cured particles on the inclined surface at low inclination angles. The surface area cleaned (dust mitigated) on the glass samples remains larger for the solution cured dust particles as compared to that of the untreated dust particles. This magnifies as the avalanche effect is introduced for dust mitigation. The interlocking of particles contributes to the bulk mitigation of the solution cured dust on the inclined glass surface. The dust layer (~ 50 µm) reduces the optical transmittance of the glass by almost over 50%. Dust mitigation under avalanche influence regains the optical transmittance of the dusty glass and the transmittance difference between the glass and dust mitigated surface is almost 1%, which shows significant improvement in regaining the optical transmittance of the glass. Hence, mitigation of the solution cured dust particles on the glass surface under the avalanche influence significantly cleans the glass surface and improves the optical transmittance.
